# Esophageal Cancers: A Clinicopathologic and Immunohistochemical Study of 223 Cases

**DOI:** 10.4021/gr2009.05.1293

**Published:** 2009-05-20

**Authors:** Tadashi Terada

**Affiliations:** Department of Pathology, Shizuoka City Shimizu Hospital, Miyakami 1231 Shimizu-Ku, Shizuoka 424-8636, Japan. E-mail: piyo0111jp@yahoo.co.jp

**Keywords:** Esophagus, Benign lesions, Malignant lesions, Clonicopathologies, Immunohistochemistry

## Abstract

The author reviewed 950 cases of consecutive esophageal biopsies in the last 15 years in out pathology laboratory of our hospital. There were 223 malignant lesions (23.5%). The number and frequency (percentages) were as follows: 54 mild dysplasia (5.7%), 30 moderate dyplasia (3.2%), 32 severe dysplasia (3.4%), 13 carcinoma in situ (1.4%), 71 squamous cell carcinoma (7.5%), 7 primary adenocarcinoma (0.7%), 1 primary signet ring cell carcinoma (0.1%), 4 primary small cell carcinoma (0.4%), 2 primary amelanotic malignant melanoma (0.2%), 1 primary undifferentiated sarcoma (0.1%), 7 gastric cancer invasion (0.7%), and 1 primary adenoid cystic carcinoma (0.1%). In this article, the clinical, histopathologic and immunohistochemical features of these esophageal cancers were described.

## Introduction

Many kinds of pathologic lesions occur in the esophagus. They include esophageal atresia, heterotopic gastric mucosa, heterotopic pancreatic tissue, diverticula, esophageal cyst, achalasia, Lye stricture, reflex esophagitis, Barrett’s esophagus, dysplasia and carcinoma in Barrett’s esophagus, Herpes simplex esophagistis, cytomegalovirus esophagitis, eosiphophilic esophagitis, Crohn’s disease, candidiasis, squamous cell carcinoma, carcinoma in situ, intraepithelial neoplasm (dysplasia), sarcomatoid carcinoma, verrucous carcinoma, adenocarcinoma, adenosquamous carcinoma, mucoepidermoid carcinoma, basaloid carcinoma, small cell carcinoma, leiomyoma, leiomyosarcoma, gastrointestinal stromal tumor, carcinoid tumor, lymphoepithelioma-like carcinoma, glycogenic acanthosis, amyloidosis, squamous papilloma, hyperplastic polyp, granular cell tumor, malignant melanoma, malignant lymphoma, plasmacytoma, malignant mesenchymal tumors, and metastatic carcinoma [1, 2]. Recent advances in endoscopy have made it possible that these esophageal lesions are biopsied and diagnosed correctly. In the present study, the authors reviewed 950 archival cases of the esophageal biopsies in search for esophageal cancers.

## Materials and Methods

The authors retrospectively reviewed consecutive 950 cases of esophageal biopsy specimens in the last 15 years in the pathology laboratory in our hospital. In the person with multiple biopsies, the number of biopsy was counted as one. The ages of the patients ranged from 12 years to 95 years with a mean of 53 years. Male to female ratio was 556:394. Clinical and endoscopic records were also reviewed.

Histochemical stainings including PAS and alcian blue were employed in appropriate biopsies. In appropriated cases, an immunohistochimical study was performed, using Dako Envision method (Dako Corp., Glostrup, Denmark), as previously described [3, 4]. The antibodies employed were anti-cytokeratin (AE1/3, Dako), anti-cytokeratin (polyclonal wide, Dako), anti-p53 protein (DO-7, Dako) anti-Ki-67 antigen (MIB-1, Dako), CD3 (M7193, Dako), CD10 (M0727, Dako), CD15 (M0733, Dako), CD30 (M0751, Dako), CD45 (M0855, DAKO), CD45RO (M0834, Dako), CD79α (M7050, Dako), CD56 (MOC-1, Dako), carcinoembrionic antigen (polyclonal, Dako), chromorgranin (DAK-A3, Dako), synaptophysin (polyclonal, Dako), neuron-specific enolase (BBS/NC/VI-H14, Dako), KIT (polyclonal Dako), PDGFRA (polyclonal, Santa Cruz, CA, USA), CD34 (QBEND10, Dako), vimentin (Vim 3B4, Dako), desmin (D33, Dako), α-smooth muscle actin (1A4, Dako), S100 protein (polyclonal, Dako), myoglobin (polyclonal), and melanosome (HMB45, Dako).

## Results

**Figure 1:**
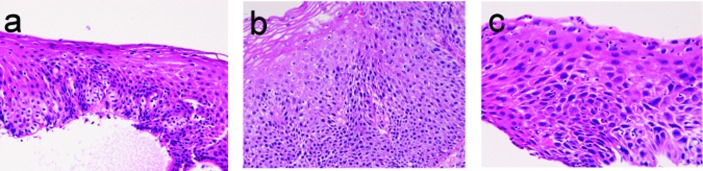
Dysplasia lesions of esophagus. (a) mild dysplasia; (b) moderate dysplasia; (c) severe dysplasia. HE X200.

**Figure 2:**
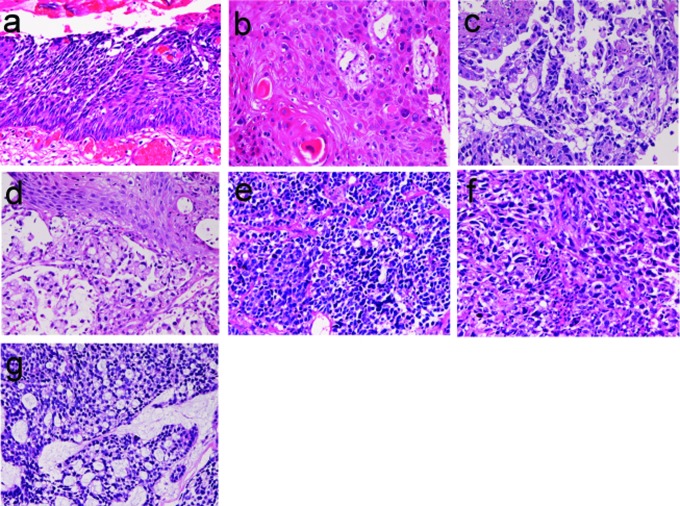
Malignant lesions of esophague. (a) carcinoma in situ of the esophagus; (b) squamous cell carcinoma of the esophagus; (c) ordinary adenocarcinoma of the esophagus; (d) signet ring cell carcinoma of the esophagus; (e) small cell carcinoma of the esophagus; (f) amelanotic melanoma of the esophagus; (g) adenoid cystic carcinoma of the esophagus. HE, X100.

There were 223 cancers (23.8%) among the 950 esophageal specimens. Mild dysplasia (Fig. 1a) was identified in 54 cases (5.7%); in the proximal esophagus in 14 cases, in the middle esophagus 18 cases, in the distal esophagus in 22 cases. Moderate dysplasia (Fig. 1b) was recognized in 30 cases (3.2%); in the cervical esophagus in 2 cases, in the proximal esophagus in 8 cases, in the middle esophagus in 9 cases and in the distal esophagus in 11 cases. Severe dysplasia (Fig. 1c) was seen in 32 cases (3.4%); in the cervical esophagus in 2 cases, in the proximal esophagus in 8 cases, in the middle esophagus in 10 cases, and in the distal esophagus in 12 cases. Carcinoma in situ (Fig. 2a) was present in 13 cases (1.4%); in the middle esophagus in 5 cases and in the distal esophagus in 8 cases. Immunohistochemically, p53 protein expression was 21/54 in mild dysplasia, 24/30 in moderate dysplasia, 30/31 (32?) in severe dysplasia, and 13/13 in carcinoma in situ. Mean Ki-67 labeling was 8% in mild dysplasia, 13 % in moderate dysplasia, 23 % in severe dysplasia, and 36 % in carcinoma in situ. These intraepithelial neoplasms were endoscopically recognized as flat, elevated, or depressed iodine-negative areas.

Squamous cell carcinoma (Fig. 2b) was recognized 71 cases (7.5%); in the cervical esophagus in 1 case, in the distal esophagus in 9 cases, in the middle esophagus in 21 cases, and in the distal esophagus in 40 cases. Of the 71 cases, 24 were well, 26 were moderately, and 21 were poorly differentiated squamous cell carcinomas. Of the 71 cases, 56 cases showed dysplasia or carcinoma in situ in the vicinity of squamous cell carcinoma. Expression of p53 protein was recognized in 69/71 cases, and mean Ki-67 labeling was 58%. Endoscopically, it was identified as a polypoid, elevated or ulcerated tumor. Primary ordinary adenocarcinoma (Fig. 2c) was demonstrated 7 cases (0.8%); in the proximal esophagus in 1 case, in the middle esophagus in 4 cases, and in the distal esophagus in the 2 cases. Of the 7 cases, 3 were well, 2 were moderately and 2 were poorly differentiated adenocarcinomas. Immunohistochemically, carcinoembrionic antigen and p53 protein were positive in all cases. Mean Ki-67 labeling was 67%. Primary signet ring cell carcinoma (Fig. 2d) was noted in 1 case (0.1%) in the middle esophagus. It contained acidic and neutral mucins, and immunohistochemically positive for cytokeratins and p53 protein. The Ki-67 labeling was 29%. These primary adenocarcinoma cases were endoscopically recognized in elevated or ulcerated tumors.

Primary small cell carcinoma (Fig. 2e) was identified in 4 cases (0.4%); in the proximal esophagus in 1 case, in the middle esophagus in 2 cases, and in the distal esophagus in 1 case. Immunohistochemically, small cell carcinoma was positive for cytokeratin and also positive for at least one of neuroendocrine markers (neuron-specific enolase, chromogranin, synaptophysin, and CD56). All cases were immunoreactive for KIT and PDGFRA. Small cell carcinoma was negative for lymphoma markers such as CD3, CD20 and CD45. It was endoscopically demonstrated as a polypoid tumor. Malignant melanoma (Fig. 2f) was present in 2 cases (0.2%), in the middle esophagus in both cases. The melanomas were amelanotic, and immunohistocheically positive for melanosome and S100 protein. Both cases were endoscopically recognized as polypoid lesions. Primary undifferentiated sarcoma was seen in 1 case (0.1%) in the cervical esophagus. The sarcoma was immunohistochemically positive for vimentin but negative for cytokeratins and other mesenchymal markers. Histologically, no specific patterns were recognized; therefore the exact diagnosis was impossible. Invasion from gastric adenocarcinoma was seen in 7 cases (0.8%) in the distal esophagus. Adenoid cystic carcinoma (Fig. 2g) was identified as elevated lesion in the middle esophagus in 1 case (0.1%).

Immunohistochemically, tumor cells were positive for cytokeratins, p53 protein and smooth muscle actin. The Ki-67 labeling was 26%.

## Discusson

In preneoplastic and malignant lesions, it is well established that mild dysplasia and moderate dysplasia were classified as low-grade intraepithelial neoplasm, and severe dysplasia and carcinoma in situ as high-grade intraepithelial neoplasm [1, 2]. The intraepithelial neoplasm may evolve into squamous cell carcinoma, and patients with this lesion have 30-60 fold increase for the development of squamous cell carcinoma [2].

In the present study, mild dysplasia was identified in 54 cases (5.7%), moderate dysplasia in 30 cases (3.2%), severe dysplasia in 32 cases (3.4%), and carcinoma in situ in 13 cases (1.4%). The frequent presence of dysplasia in the vicinity of squamous cell carcinoma in the present study highly suggests that these dysplastic lesions lead to the development of squamous cell carcinoma. The choice of therapy is endoscopic mucosal resection.

As is well known, squamous cell carcinoma is the most common aggressive tumor in the esophagus. In the present study, squamous cell carcinoma was recognized 71 cases (7.5%); it occured preferentially middle and distal esophagus. The frequent p53 expression and high Ki-67 labeling suggest that p53 gene is mutated and proliferative fraction is very high. Primary ordinary adenocarcinoma is a very rare entity. It was demonstrated in 7 cases (0.8%) in the present study. Primary signet ring cell carcinoma is an extremely rare tumor in the esophagus [5]. In the present study, it was recognized in only 1 case (0.1%).

Primary small cell carcinoma is a very rare neoplasm in the esophagus [6]. In the present series, it was identified in 4 cases (0.4%). Immunohistochemically, it was characterized by positive neuroendocrine markers and positive KIT and PDGFRA. The latter two were new findings. Primary malignant melanoma is also an extremely rare neoplasm in the esophagus [7]. In the present series, it was recognized in 2 cases (0.2%). Both cases were amelanotic melanomas. In the present series, undifferentiated sarcoma was seen in 1 case (0.1%). The cell type of the sarcoma was indeterminate despite of various immunohistochemical procedures. Adenoid cystic carcinoma is very rare in the esophagus [1, 2, 8]. It is believed to originate from esophageal glands [1, 2]. In the present series, it was identified in only 1 case (0.1%). In the present series, invasion from gastric adenocarcinoma was seen in 7 cases (0.8%). It must be differentiated from primary adenocarcinoma of the esophagus.
